# Long-Term Survival of Human Neural Stem Cells in the Ischemic Rat Brain upon Transient Immunosuppression

**DOI:** 10.1371/journal.pone.0014035

**Published:** 2010-11-19

**Authors:** Laura Rota Nodari, Daniela Ferrari, Fabrizio Giani, Mario Bossi, Virginia Rodriguez-Menendez, Giovanni Tredici, Domenico Delia, Angelo Luigi Vescovi, Lidia De Filippis

**Affiliations:** 1 Department of Biotechnologies and Biosciences, University Milano Bicocca, Milan, Italy; 2 Department of Neurosciences and Biomedical Technologies, University Milano Bicocca, Milan, Italy; 3 Department of Experimental Oncology, Fondazione IRCSS Istituto Nazionale Tumori, Milan, Italy; 4 IRCCS Casa Sollievo della Sofferenza, Opera di San Pio da Pietralcina, San Giovanni Rotondo, Italy; University of North Dakota, United States of America

## Abstract

Understanding the physiology of human neural stem cells (hNSCs) in the context of cell therapy for neurodegenerative disorders is of paramount importance, yet large-scale studies are hampered by the slow-expansion rate of these cells. To overcome this issue, we previously established immortal, non-transformed, telencephalic-diencephalic hNSCs (IhNSCs) from the fetal brain. Here, we investigated the fate of these IhNSC's immediate progeny (i.e. neural progenitors; IhNSC-Ps) upon unilateral implantation into the corpus callosum or the hippocampal fissure of adult rat brain, 3 days after global ischemic injury. One month after grafting, approximately one fifth of the IhNSC-Ps had survived and migrated through the corpus callosum, into the cortex or throughout the dentate gyrus of the hippocampus. By the fourth month, they had reached the ipsilateral subventricular zone, CA1-3 hippocampal layers and the controlateral hemisphere. Notably, these results could be accomplished using transient immunosuppression, i.e administering cyclosporine for 15 days following the ischemic event. Furthermore, a concomitant reduction of reactive microglia (Iba1+ cells) and of glial, GFAP+ cells was also observed in the ipsilateral hemisphere as compared to the controlateral one. IhNSC-Ps were not tumorigenic and, upon in vivo engraftment, underwent differentiation into GFAP+ astrocytes, and β-tubulinIII+ or MAP2+ neurons, which displayed GABAergic and GLUTAmatergic markers. Electron microscopy analysis pointed to the formation of mature synaptic contacts between host and donor-derived neurons, showing the full maturation of the IhNSC-P-derived neurons and their likely functional integration into the host tissue. Thus, IhNSC-Ps possess long-term survival and engraftment capacity upon transplantation into the globally injured ischemic brain, into which they can integrate and mature into neurons, even under mild, transient immunosuppressive conditions. Most notably, transplanted IhNSC-P can significantly dampen the inflammatory response in the lesioned host brain. This work further supports hNSCs as a reliable and safe source of cells for transplantation therapy in neurodegenerative disorders.

## Introduction

The isolation of multipotent neural stem cells (NSCs) from the human central nervous system (CNS) has spurred the investigation of new cell-therapy approaches for brain injuries and neurodegenerative diseases. NSCs, which reside in specialized regions of the adult CNS, in particular in the subventricular zone (SVZ) [1]–[3] and the dentate gyrus of the hippocampus (DG), possess life-long self-renewal and the ability to generate neurons, astrocytes and oligodendrocytes. Although NSCs play a central role in CNS development and cellular homeostasis throughout adulthood [2], [4], [5], limited spontaneous recovery is known to occur following brain damage [6], [7]. Nonetheless, the integration of functional new neurons following injury can be achieved by the mobilization of endogenous stem cells [8], [9] or by transplanting new cells from different sources, as shown in experimental models of ischemia [10]–[12].

Also owing to the resilience of hNSCs (human neural stem cells) to expansion ex vivo, a relatively limited number of studies has investigated the use of hNSCs for the experimental treatment of cerebral ischemia [13]. An initial solution to this issue has come from the establishment of non-transformed, v-myc immortalized hNSCs, to give rise to stable cell lines (IhNSCs) [14], that can be rapidly expanded *in vitro* and retains the features of parental NSCs, such as proliferation, self-renewal, functional stability and multipotency.

In this paper, we demonstrate that the IhNSC's immediate progeny, represented by neural progenitors undergoing early differentiation phases (IhNSC-Ps) exhibit widespread integration ability and long-term survival when transplanted into the brain of adult rats lesioned by transient global ischemia. IhNSC-Ps generated both glial cells and mature neurons, both in the cortex and the corpus callosum. We also found that IhNSC-P-derived neuronal cells were able to establish heterotypic synaptic junctions with the host tissue after 4 months from transplantation.

Although several studies have reported a weak host' immunogenic response against transplanted hNSCs and their progeny in the brain, this issue has never been unraveled [15]–[18]. Thus, we investigated the immunogenic response of our immortal hNSCs' progeny and were able to show that grafted IhNSC-Ps have the ability to integrate in the post-ischemic, inflammatory environment that develops in the brain after injury, also dampening the local inflammatory reaction at the integration sites. All of the above was accomplished even using transient immunosuppression.

## Materials and Methods

### Transient Global Ischemia

All animal experimental protocols were approved by the Ethics Review Committee for Animal Experimentation of the Italian Ministry of Health (protocol number 37/2007-B). Adult male Sprague-Dawley rats (350–400 gr) were anesthetized with ketamine (60 mg/Kg) and Xylazine (10 mg/Kg). The common carotid arteries were exposed bilaterally by means of a ventral midline incision and occluded with microvascular clips for 10 minutes. The body temperature of the rats was mantained at 37°±0.5°C by a heating pad provided with a rectal probe. All physiological parameters were monitored and recorded throughout the surgery with BIOPAC Data Acquisition System. During the 10 minutes of carotid occlusion, mean blood pressure was maintained at 50 mmHg by withdrawal of blood from the femoral artery previously exsposed and incannulated with PE50 tubing connected to a BIOPAC system and to a collector. After the removal of the clips from the carotid arteries, the blood was reinjected into the femoral artery. After the surgery, the rats were daily treated with subcutaneous injections of antibiotics (Enrofloxacin 10–15 mg/Kg) and painkillers (Carprofen 5 mg/Kg) for one week.

### Cell Preparation

To generate IhNSC-Ps for transplantation, IhNSC neurospheres, cultured as described in De Filippis et al. 2007, were mechanically dissociated and transferred onto laminin (Roche, Base, Switzerland, http://www.roche-applied-science.com)- coated tissue culture flasks (or glass coverslips for immunostaining assays) at a density of 1×10^4^ cells per cm^2^ in the presence of FGF2 (20 ng/ml) for 3 days. The day of transplant IhNSC-Ps were collected with VERSENE (Gibco, Aukland, NZ) and transferred into control medium ad the density of 1×10^5^ cells/µL.

Characterization was performed by immunostaining assays with primary antibodies β-TubulinIII (β-Tub, TUJ-1, 1∶400, Covance), Galactocerebroside C (GalC, 1∶100, Chemicon), Glial fibrillary acidic protein (GFAP, 1∶500, Chemicon), Green Fluorescent Protein (GFP, 1∶500, Sigma), Microtubule-associated protein 2 (MAP2, 1∶200, Sigma), Doublecortin (DCX, 1∶200, Santa Cruz) and Neural Cell Adhesion Molecule (NCAM, 1∶100, Santa Cruz).

### Transduction of IhNSC with lenti-gfp

Transduction of IhNSC with a lentiviral vector carrying the gfp gene was carried as described in [19], [20] and the percentage of GFP+ IhNSC reached 95%. After 4 passages they were used for transplantation.

### Cell Transplantation

Experimental design (Figure S3) included the following animal groups: healthy control animals (n = 4), healthy control animals transplanted with IhNSC-Ps in the corpus callosum (n = 2/each time point), healthy control animals transplanted with IhNSC-Ps in the hippocampal fissure (n = 2/each time point) and lesioned animals transplanted with IhNSC-Ps (n = 2/each time point) and GFP+ IhNSC-Ps cells (n = 3/each time point) [19] in the corpus callosum (n = 5 tot/each time point) or in the hippocampal fissure (n = 5 tot/each time point). The four groups of transplanted animals were transiently immunosuppressed with cyclosporine (see below) and sacrificed at 7 days, 2 weeks, 1 month, 3 and 4 months from transplantation and analyzed in parallel with healthy controls.

In the paralel a set of lesioned animals was transplanted with GFP+ IhNSC-Ps in the periventricular region next to cc (n = 3 each time point) or in the hippocampal fissure (n = 3 each time point), constitutively immunosuppressed and sacrificed 1, 3 and 4 months later.

Rats were anesthetized with an intraperitoneal injection of ketamine (60 mg/Kg) and Xylazine (10 mg/Kg), placed in a sterotactic frame (David Kopf Instruments, Tujunga, CA) and injected with 2 µL of cell suspension (1×10^5^ cells/µL control medium) using a Hamilton syringe to the hippocampal fissure (anteroposterior: −5.3; lateral: +3.0; dorsoventral: −3.0) or to the posterior periventricular region in the cc (anteroposterior: −5.3; lateral, +3; dorsoventral: −2). All animals were immunosoppressed with Cyclosporine A (15 mg/Kg; Sandimmun, Novartis) administered subcutaneously starting 2 days before transplantation and for the duration of the study or for 14 days for transient immunosuppression experiments.

### Tissue Processing and Immunohistochemistry

Rats were anestethized with an intraperitoneal injection of Avertin (300 mg/Kg) and transcardially perfused-fixed with 4% paraformaldehyde. Brains were fixed overnight in 4% paraformaldeyde at 4°C, then sequentially transferred in 10%, 20% and 30% sucrose solutions. Brains were then cryopreserved (Killik, Bio-Optica, Italy), frozen and stored at −80°C. Coronal sections (18 µm thick) were obtained using a cryostat, transferred onto Super Frost/Plus object glasses (Menzel-Glaser, Braunschweig, Germany) and stored at −20°C. Sections were let dry at room temperature for 1 hour, rehydrated in phosfate-buffered saline and blocked with phosphate-buffered saline containing 10% Normal Goat Serum and 0,3% Triton X-100 for 90 minutes at room temperature. The following primary antibodies and dilutions were used: Human Specific Nuclei (HuN, 1∶100, Chemicon), β-TubulinIII (TUJ-1, 1∶400, Covance), Gamma-aminobutyric acid (GABA, 1∶500, Sigma), Glial fibrillary acidic protein (GFAP, 1∶500, Chemicon), Green Fluorescent Protein (GFP, 1∶500, Sigma), Glutamate (GLUTA, 1∶500, Sigma), Microtubule-associated protein 2 (MAP2, 1∶200, Sigma), Doublecortin (DCX, 1∶200, Santa Cruz), Neural Cell Adhesion Molecule (NCAM, 1∶100, Santa Cruz), human specific Ki67 (Ki67, 1∶200, Novocastra), Iba1 (1∶100, Wako). The fluorescent secondary antibodies used were labelled with Cy3 (1∶800, Jackson), Cy2 (1∶200, Jackson), Alexa Fluor 546 and 488 (1∶800, Molecular Probes). DAPI (ROCHE) was used as nuclear marker. Immunofluorescence-labeled sections were viewed under a fluorescence microscope (Zeiss Axioplan 2 imaging) and a confocal microscope (Leica Dmire2).

### Quantification of cell death in the CA1 layer, survival of transplanted cells and micro/astroglial cells

The percentage of dying cells was assessed by counting the pyknotic nuclei over total nuclei into the CA1 layer of lesioned and healthy control animals in serial brain sections (each 200 µm) as described below (n = 3 rats/time point).

At different time points, the rate of survival of IhNSC-Ps was evaluated by counting GFP+ or HuN+ cells in serial brain sections (each 200 µm apart) spanning the graft area of n = 3 rats per time point. The total number of surviving transplanted cells was calculated for the whole graft using Abercrombie formula [21]. Data is presented as the percentage of surviving cells over total transplanted cells (200.000), calculated as the mean average among the animals of each experimental group.

The percentage of Iba1+ or GFAP+ cells was counted by sampling three field in the hippocampal region of healthy or lesioned rats at 3, 7, 14 and 30DAI.

For all the quantifications an average number of 3 sections was counted per rat spanning about 500 µm along the antero-posterior axis.

Statistical analysis was performed by one-way ANOVA. Data is reported as means±SEM. (*P<0.05. **P<0.01, ***P<0.001).

### GFP immunolabeling and electron microscopy

Animals were perfused with 4% paraformaldehyde in phosphate buffer (0.12 M, pH 7.4). Brain samples were then cut using a vibratome (section thickness 30–40 µm). Free-floating brain slices were washed in Tris-buffered saline (TBS) and pre-incubated in 3% goat normal serum (NGS) in TBS for 30 min. Cells were also fixed with 4% formaldehyde and rinsed in TBS. Sections and cells were subsequently incubated with a rabbit anti-GFP antibody (1∶250 for brain slices, 1∶650 for cell cultures; Chemicon International) overnight at 4°C in 1% NGS/TBS. A secondary antibody (goat anti-rabbit HRP-labeled, 1∶250 dilution; PerkinElmer, Boston, MA) was used for 1 h at room temperature before developing the immunoreactive signal determined by the reaction of 3,3′-diaminobenzidine tetrahydrochloride (DAB; Sigma, St. Louis, MO) with H_2_O_2_.

Immunolabeled samples were post-fixed in 1% OsO_4_ in cacodylate buffer (0.12 M, pH 7.4) for 45–60 min, dehydrated and embedded in Epoxy resin. Ultramicrotome (Ultracut E, Reichert-Jung) 60 nm sections (both rat hippocampus and cultured cells) were then examined by a Philips CM 10 transmission electron microscope. Images were taken with a Mega View II digital camera (Soft Imaging System).

## Results

**Figure 1:**
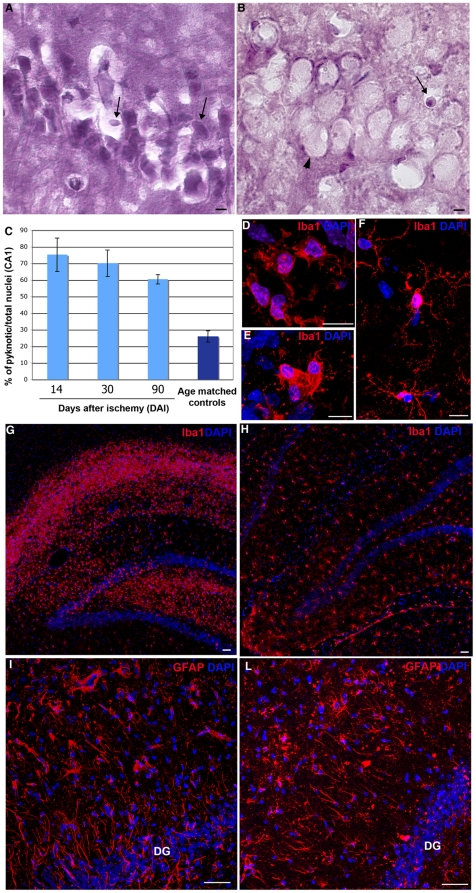
Neuronal loss and inflammation in the ischemic hippocampus. (A–B) Damaged cells at 14DAI (pyknotic nuclei, arrows) versus normal CA1 cells (B arrowhead). (C) Quantification of damaged cells in control and lesioned animals. (D–F) Iba1+ microglial cells with ameboid (lesioned hippocampus D–E) or stellate (control F) morphology. (G–H) Increase of microglia (Iba1+ cells) in the lesioned hippocampus (G, 7DAI) with respect to control (H). (I–L) Morphology of astroglial cells in the lesioned hippocampus (I, 7DAI) and control (L). Scale bars: A, B: 5 µm, D, E and F: 10 µm, G, H, I and L: 50 µm.

We have previously shown that IhNSC transplantated into the immunodficient SCID mice brain can survive for as long as 6 months [14]. Nonetheless, pre-clinical and, most important, clinical neural transplantation are based on the concept that continuous immunosuppressive treatments are to be used to avoid donor cell rejection [18]. In addition, the possibility that the stroke heavy inflammatory environment (see Fig. 1) might enhance cell rejection compounds the problem further. In this view, we performed experiments in which animals transplanted with GFP-expressing IhNSC-Ps were treated with cyclosporine, either continuously (starting on the first day after ischemia (DAI 1) till the end of the experiment) or only transiently, i.e. for only two weeks, starting on DAI 1 and finishing on DAI 14. Tissues were analyzed 1, 3 and 4 months after implantation. Much to our surprise, no significant differences in the survival or integration were detected in transiently immunosuppressed animals (56.7%; n = 17/30) as compared to those receiving cyclosporine for the whole duration of the experiments (66.7%; n = 10/15). In considering that the two types of immunosuppressive protocols yielded overlapping results, the data presented below refer to milder one, i.e the transient administration of cyclosporine administered in the peri-transplantation period (two days before cell injection, all the way to 12 days after the latter took place).

### Evaluation of the ischemic lesion

The lesion generated by transient global ischemia in the central nervous system is widespread and involves most brain districts. Notwithstanding, cortical areas and the CA1 layer of the hippocampus (Figure S1A–C) are known to be the most affected by this type of injury [22], [23]. In this view, in order to assess the ability of I-hNSC-P to integrate following ischemic brain tissue damage, we focused our study on the hippocampal region and standardized our investigation by quantifying the fraction of pyknotic nuclei in the CA1 layer, as detected by hematoxilin-eosin staining (Fig. 1A and B and Figure S1C). Therein, the fraction of dead cells amounted to approximately 75% of the total CA1 cells at 3 and 14 DAI, with their number progressively decreasing to 60% at 90 DAI, as compared to the physiological 25% fraction in control animals (Fig. 1C and Figure S1F). Given the inflammatory nature of this kind of ischemic injury, we also investigated the activation of microglia and astroglia during the subacute phase of the lesion, that is at 3 days (Figure S1D–F) and 1 week from ischemia: no significative changes in the inflammatory environment were detected between these two time points. In the lesioned brain, Iba1+ cells presenting an ameboid morphology, typical of reactive, macrophagic microglia (Fig. 1D and E) – in sharp contrast with the star-shaped resting microglia found in the non-lesioned brain (Fig. 1F) – were dramatically increased both in the CA1 layer and in the hilus of the dentate gyrus (Fig. 1G) as compared to control animals (Fig. 1H). This was consistent with the data concerning the analysis of reactive gliosis by GFAP immunostaining, showing a striking alteration of astrocyte morphology (Fig. 1I) characterized by thicker and shorter processes in the lesioned brain as compared the healthy controls (Fig. 1L).

### IhNSC-Ps efficiently survive after transplantation into the ischemic rat brain

In order to contribute to the neural regeneration in the early phases following tissue damage, IhNSC-Ps cells were transplanted nearby the CA1 layer soon after lesioning. Previous results with various transplantation paradigms in several animal models have shown that transplantation of undifferentiated NSCs cells from neurospheres generate mainly glial progenitors upon engraftment [20], [24] or remain undifferentiated in vivo [25], [26]. Hence, we decided to commit the IhNSCs' progeny in vitro, prior to implantation. To do this, we pre-differentiated IhNSCs for 3 days in the presence of FGF2 in order to induce early neuronal progenitors' proliferation and to favour their fate choice towards the neuronal lineage [27], [28]. By this, the IhNSC-Ps used for transplantation contained cells of the neuronal lineage, that expressed both the early markers Dcx and NCAM (21.97±4.21% and 21.11±4.5%, of the total differentiated cells, respectively; Figure S2A, B and F), and the late markers β-Tub (13.37±2.71%, Figure S2C and F) and MAP2 (6.12±4.1%, Figure S2D and F), as well as astroglial GFAP+ (15.91±3.91% Figure S2C and F) and oligodendroglial GalC+ (8.6±2.65%, Figure S2E and F) cells. This was quite different from IhNSC cultured in the presence of EGF and FGF2 (neurospheres) which, in turn, contained only sporadic β-Tub+ cells and low percentages of MAP2+ (3.57±1.75%) and GalC+ (5.25±0.77%) cells. As expected for a population intended to contain early transient dividing progenitors, most IhNSC-Ps were actively proliferating, with 71.1±6.6% (Figure S2F) being positive for Ki67 (Figure S2A, B and D), as compared to 46.4±3.5% in undifferentiated IhNSC.

**Figure 2:**
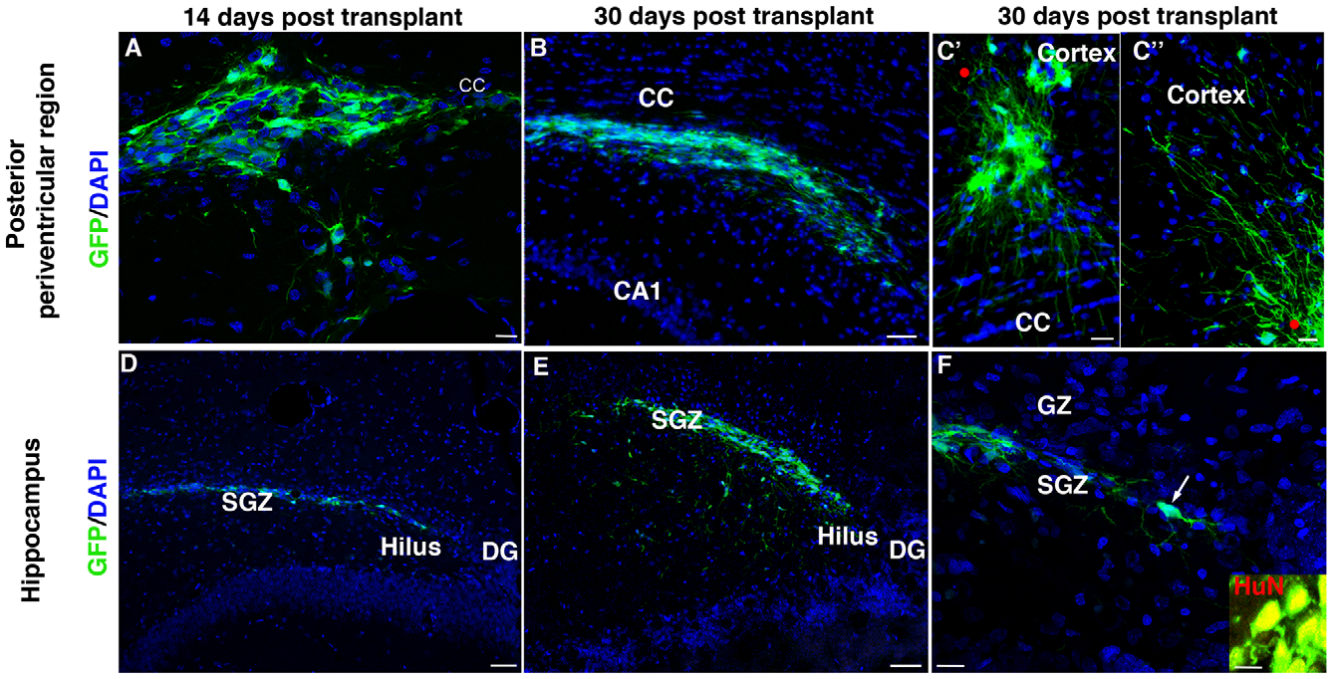
GFP-IhNSC-Ps survive in the ischemic brain. (A–C″) IhNSC-Ps into the cc at 14 (A) and 30 (B) days post transplant. Long processes from donor cells directed toward the cc (C′) and the upper cortical layer (C″). (D–F) Distribution of IhNSC-Ps along the SGZ at 14 days (D) and migrating to the lower SGZ at 30 days (E). Single GFP-IhNSC-Ps with tipical stem cell phenotype in the SGZ (F, arrow). Confocal analysis of colocalization of HuN with GFP (inset in F). cc: corpus callosum, GZ: granular zone, SGZ: subgranular zone, DG: dentate gyrus. Scale bars: A, F, F inset: 10 µm, C′ and C″: 20 µm, B, D, E: 50 µm.

The IhNSC-Ps were injected into the posterior periventricular region, next to the corpus callosum or in the hippocampal fissure of rats at 3 DAI (Figure S3). Integrated surviving cells were detected in approximately 60.6% (n = 37/61) of the transplanted, injured animals, as compared to 25% (n = 7/28) in the control animals (not lesioned) receiving the same cells. This is in agreement with previous findings showing that the presence of CNS injury is required to favor engraftment of exogenous cells in the adult brain tissue [29]. In the injured animals where engraftment was successful, the average survival rate of IhNSC-Ps was 19.5±1,4% of the total transplanted cells and remained unchanged for over 4 months. Fourteen days after transplantation into the posterior periventricular region, donor cells were found to be located close to the injection site (n = 4), (Fig. 2A). By 30 DAI, IhNSC-Ps migrated medially and laterally, along the myelin fibers of the corpus callosum and clusters of donor cells displayed long neuronal-like processes, which were directed towards the corpus callosum, with some of the cells migrating to the upper cortical layer (n = 3) (Fig. 2B and C′, C″).

At 14 DAI, IhNSC-Ps injected into the hippocampus were found to integrate into the DG (n = 3) (Fig. 2D) and in the subgranular zone (SGZ) (n = 3) (Fig. 2E and F), crossing the hilus and reaching the lower SGZ by 30 DAI. A subset of IhNSC-Ps presented a stem cell-like morphology [30], [31], with the cell body nested in the SGZ and tangential processes extending along the border of the granule cell layer and hilus (Fig. 2F, arrow). No IhNSC-P cells were detected in the contralateral hemisphere at 2 and 4 weeks after transplantation. In control (unlesioned) animals, IhNSC-Ps were confined to the injection site (data not shown). The colocalization of GFP with the human specific antigen HuN (Fig. 2F inset) confirmed the identity of these cells as donor cells.

This analysis demonstrates that IhNSCs efficiently survive in vivo and that their engraftment and migration capacities are improved in a lesioned brain, which is consistent with previous results showing that injury generates a local environment permissive for the integration of xenotransplanted cells [17], [29], [32].

### IhNSC-Ps give rise to neuronal cells in vivo

Next, we evaluated the differentiation of IhNSC-Ps into specific neuronal and glial phenotypes following transplantation into the ischemic environment by analyzing the colocalization of selective markers for neurons, astroglial and oligodendroglial cells with the anti-human specific antibody anti-huN. This was carried out on IhNSC-Ps that were not tagged with GFP, in order to rule out possible effects on their differentiation properties, as consequence of viral transduction with the GFP expression construct.

**Figure 3:**
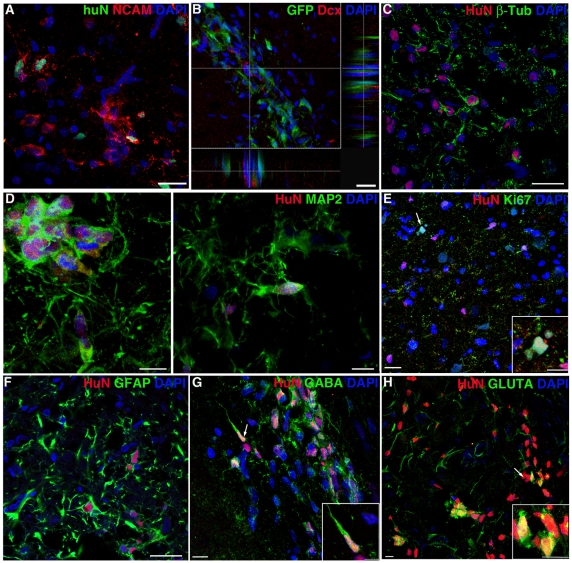
Differentiation of transplanted IhNSC-Ps in vivo. (A–F) IhNSC-Ps at 3 months post transplantation differentiate into both NCAM+HuN+ (A, cc) and Dcx+/GFP+ (B, dentate gyrus) neuronal progenitors and β-Tub+HuN+ (C) and MAP2+HuN+ (D) mature neurons in the cortex. (E) Sporadic proliferating cells (Ki67+HuN+, arrow and inset magnification). (F) GFAP+HuN+ astroglial cells in the cortex. (G–H) IhNSC-Ps at 1 month from transplantation generate GABAergic (GABA+HuN+, arrow in G and inset magnification) and Glutamatergic neurons (GLUTA+HuN+, arrow in H and magnification). Scale bars: A, B, C, E, F: 20 µm, D, inset in E, G, H, inset in H: 10 µm, inset in G: 5 µm.

At one and three months post transplantation, IhNSC-Ps migrating through the corpus callosum and localizing into the DG were found to be relatively immature neuronal cells, expressing NCAM protein (corpus callosum, Fig. 3A) or Dcx (dentate gyrus, Fig. 3B). A subset of HuN+ cells had further matured into neuronal cells expressing β-Tub+ (11.3± 0.8% over total HuN+ cells) (Fig. 3C) and MAP2+ (marker of dendritic neuronal processes), found in sporadic cell clusters or as isolated elements (Fig. 3D) within the corpus callosum and cortex. Such clusters seemed to have arisen through the in vivo, transient proliferation of single donor cells, as supported by the sporadic expression of Ki67 observed in HuN+ cells (Fig. 3E and inset). This would be in agreement with recent data reporting that ischemic injury generates in the cortex an environment favoring the proliferation of local precursors [33]. We also observed IhNSC-P-derived, stellate GFAP+/HuN+ astrocytes (29.7±3.03% over total HuN+ cells) amongst the engrafted, surviving cells (Fig. 3F), but failed in detecting oligodendroglial cells.

Altogether, the results above show that IhNSC-Ps can differentiate towards the neuronal and astroglial lineages in the ischemic brain. Both immature migratory neuroblasts and more mature β-Tub+ and MAP2+ neuronal cells are produced throughout this process.

### Neuronal subtypes derived from grafted IhNSC-Ps

We have previously shown that IhNSCs [14] can differentiate in vitro into GABAergic and Glutamatergic neurons, similar to their wild-type counterpart [34]. Therefore, we analyzed the expression of such neurotransmitters among the IhNSC-Ps' progeny that successfully engrafted in our model. We found both cells exhibiting the GABAergic (Fig. 3G and inset) and glutamatergic (Fig. 3H and inset) phenotypes in the corpus callosum and cortex, as early as 1 month after transplantation, which were still detectable 4 months from transplantation. These findings show, for the first time, that IhNSCs progeny can engraft in the adult brain as mature neuronal cells expressing the GABAergic or the glutamatergic phenotypes.

### Long-term survival of IhNSC-Ps

**Figure 4:**
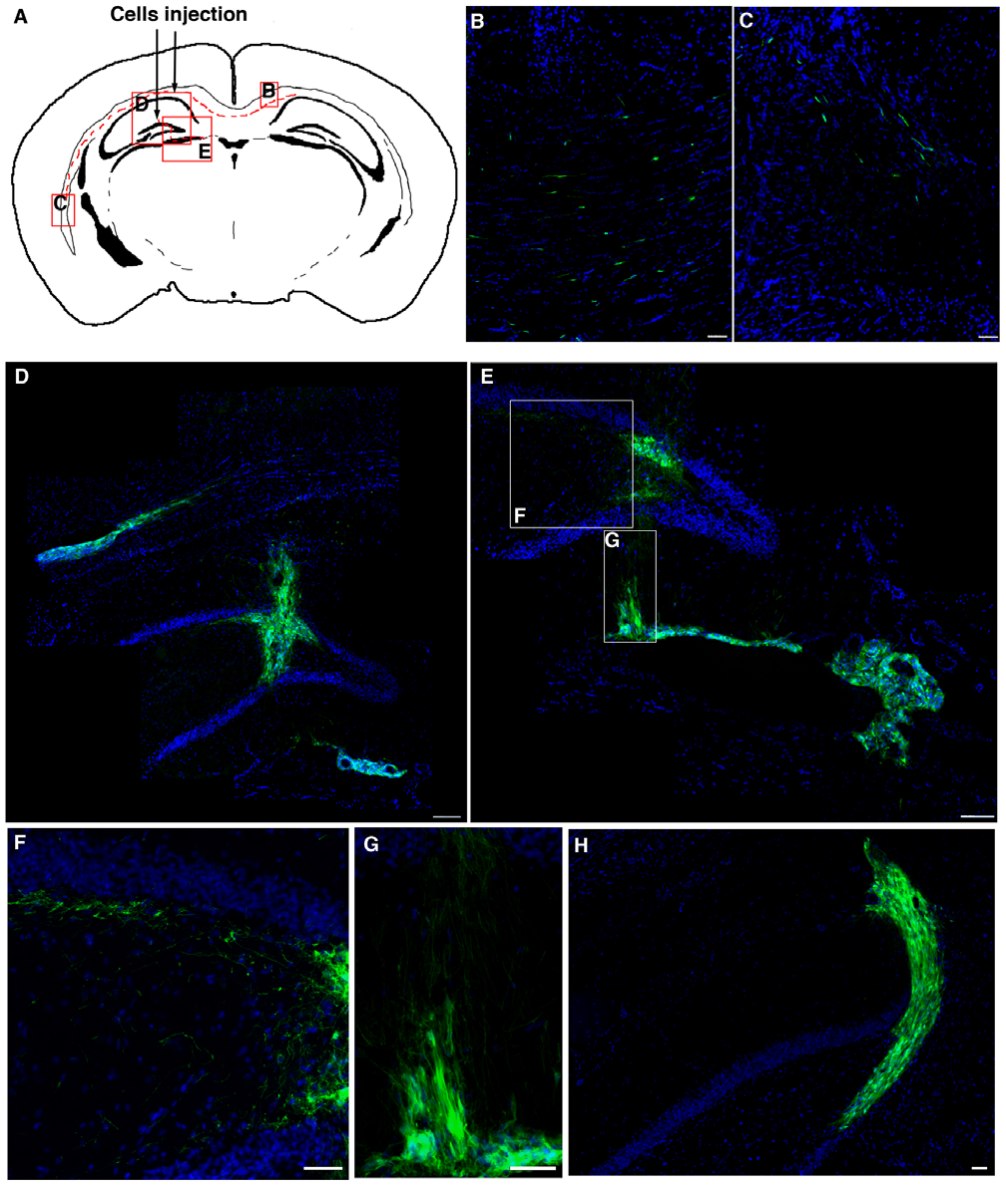
Long-term survival of IhNSC-Ps after transient immunosuppression. (A) Map of the brain areas colonized by IhNSC-Ps in transiently immunosuppressed ischemic rats 4 months following transplantation. Letters in boxed area refer to the figures B–E. IhNSC-Ps migrate extensively through the cc (B–C) and along the dentate gyrus (D, E and H) to the underling SVZ (D, E). F and G magnifications of boxed areas in E. Scale bars: B,C,E,F,G and H: 50 µm; D: 100 µm.

At the later time tested, i.e. 4 months (Fig. 4A, n = 3) the presence of IhNSC-P-derived cells was obvious in the corpus callosum, wherein they migrated tangentially (4.3±0.6 mm medially and 4.5±0.45 mm laterally, n = 3, Fig. 4B and C), also spreading into the controlateral hemisphere (Fig. 4B). GFP+ cells were also detected along the injection tract in the hippocampal fissure, along the SGZ of the dentate gyrus (n = 4) (average distance of migration 400 µm medially and 380 µm laterally to the injection site up to the SVZ, wherein they migrated 1.2±0.53 mm (n = 3) medially along the ventricles (Fig. 4D–H). These results support the concept that, immunologically, transplanted human cells are well tolerated by the adult brain and that even a quite mild immunosuppression, like the transient one used here, may be sufficient to accomplish their efficient integration in the lesioned CNS tissue.

### Migration of IhNSC-Ps' progeny and long-term integration into the CA1 layer

**Figure 5:**
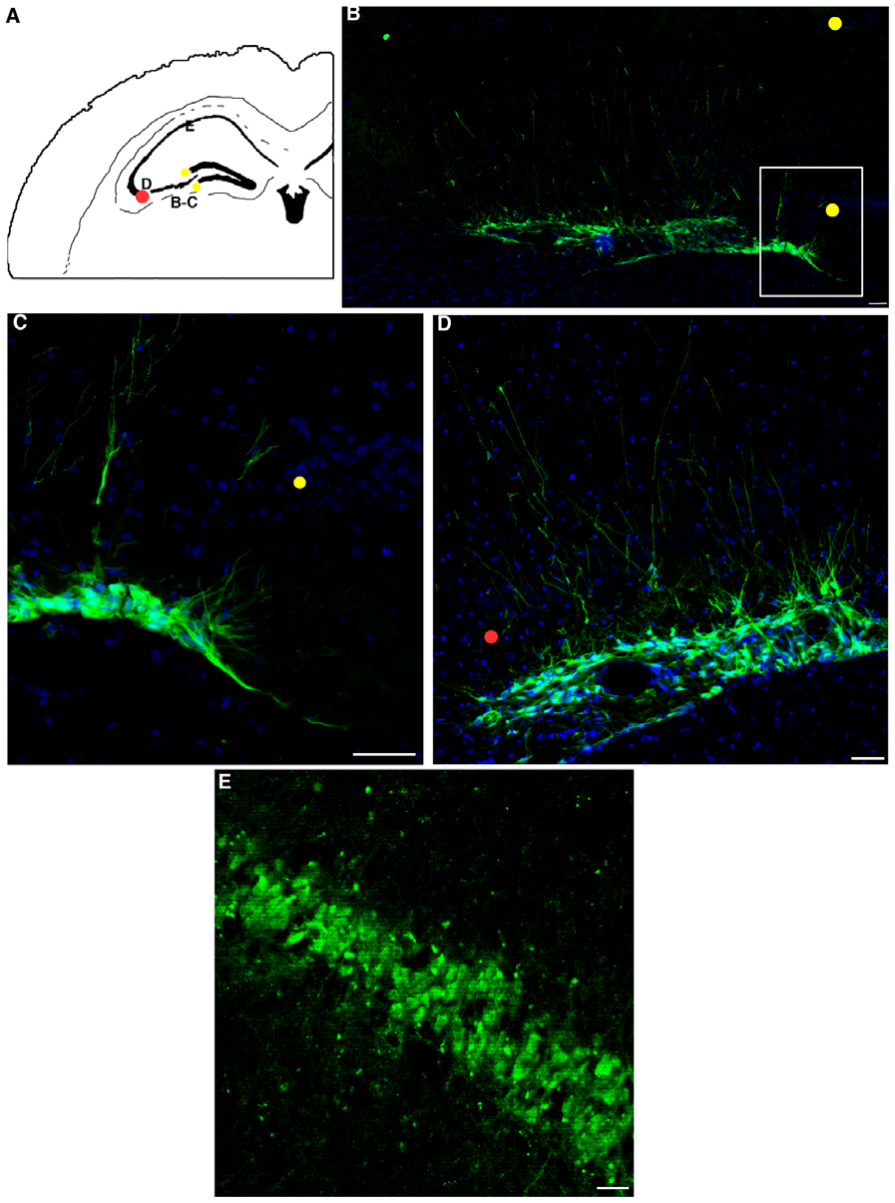
At four months after transplant IhNSC-Ps integrate in the hippocampal layers under transient immunosuppression. (A) Schematic map of the hyppocampal layers colonized by IhNSC-Ps in transiently immunosuppressed ischemic rats. The letters are positioned next to the regions referred to the figures B–E. (B–D) At 4 months following transplantation IhNSC-Ps were found integrated into the CA3 layer and in the underling SVZ (B), emitting long processes toward the dentate gyrus (boxed area in B, shown at higher magnification in (C) and CA1 layer (B–D). (E) At this time IhNSC-Ps were also found distributed along the CA1 layer. Scale bars: in B–D: 50 µm, in E: 30 µm.

The CA1 layer of the hippocampus is one of the areas most prominently damaged in transient global ischemia. In addition, neurons newly generated by adult neurogenesis in the CA1 pyramidal layer also die, due to the persistence of inflammatory conditions [35]. Consistently with these findings, we were unable to detect IhNSC-Ps in the CA1 pyramidal layer at 1, 2 or 3 months after transplantation. At 4 months after ischemia, we found that some IhNSC-Ps did integrate along the CA3 layer (n = 2), sending processes toward the CA1 layer (Fig. 5A–D) and migrating to the underlying SVZ (Fig. 5C). Unexpectedly, IhNSC-Ps were also found as irregular clusters, distributed along the CA1 layer (n = 4), with their nuclei organized according to the classic multilayer pattern of the CA1 layer (Fig. 5E). These observations show that, despite the persistence of inflammation in this region [35], IhNSC-Ps retain the ability to interact with the to host endogenous neurogenic pathways, suggesting that they can undertake appropriate differentiation and contribute to the local regeneration of the damaged hippocampal tissue.

### IhNSC-P derived neurons establish synaptic junctions in vivo

**Figure 6:**
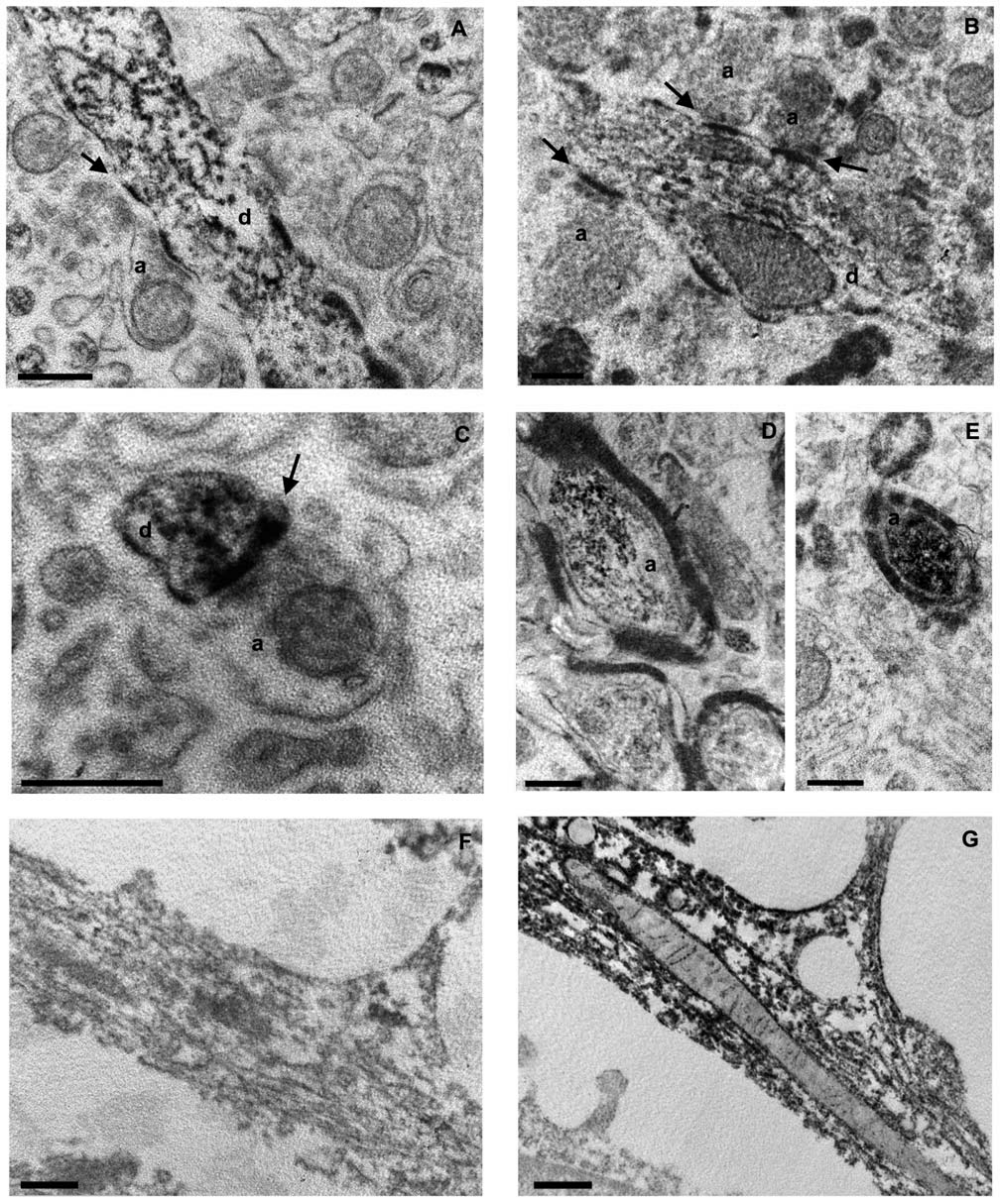
IhNSC-Ps form synapses into the rat hippocampus. Representative electron micrographs of rat hippocampus (A–E) and IhNSC-Ps differentiated *in vitro* (F–G), immunostained for GFP. (A–C) GFP immunopositive dendrites (d) on which unstained axons (a) project, making synaptic contacts (arrows). (D, E) GFP immunopositive myelinated axons (a). (F) Sham transfected IhNSC-Ps in culture. (G) GFP expressing IhNSC-Ps in culture. To note, GFP is mainly associated to microtubular structures. Scale bars: 500 nm.

Since neither electrophysiological recordings on adult rat brain slices nor high resolution immunofluorescence analysis could be performed because of the ischemia-induced decay of tissue cytoarchitecture, we assessed the ultrastuctural features of GFP-expressing IhNSC-Ps progeny located in the CA1 layer, 4 months after transplantation (Fig. 5E). In the hippocampus, GFP immunoreactivity was present within IhNSC-P-derived cell processes, mainly distributed along the cytoskeleton. Despite the disorganization of the ischemic tissue, we observed recipient axons (GFP-negative, a in Fig. 6A-C), possibly pyramidal neurons in the CA1 layer of the hippocampus, making synaptic contacts with the GFP-labeled processes (d in Fig. 6A-C). GFP-labeled myelinated axons were also detected (Fig. 6D and E). This was similar to the GFP-labeling pattern observed in cultured IhNSC-Ps, which was associated with microtubular structures (Fig. 6G) and could not be oserved in the non-transduced control cells (Fig. 6F).

This supports the notion that surviving IhNSC-Ps progeny have the ability to integrate into the lesioned CA1 area, therein establishing heterotypic synaptic junctions with host cells.

### IhNSC-Ps can modulate the inflammatory response in the post-ischemic hippocampus

**Figure 7:**
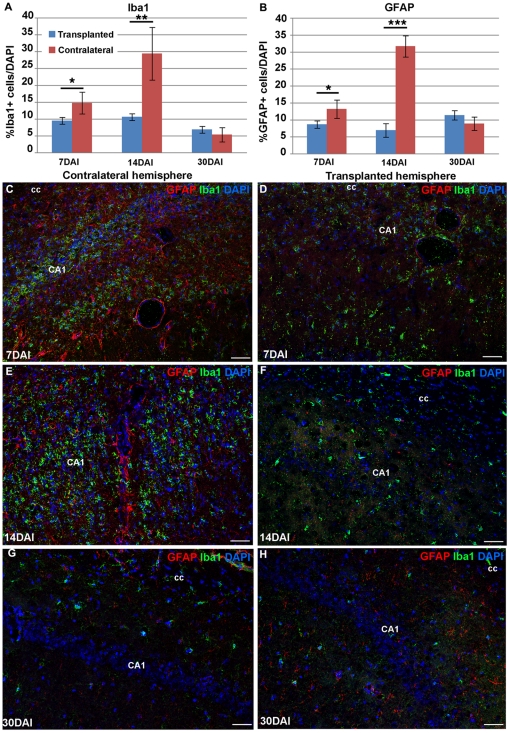
Effect of IhNSC-Ps on microglia and astrocyte activation. (A–B) Charts showing the effect of IhNSC-Ps transplantation on the number of microglia (Iba1+, A) and astroglial cells (GFAP+, B) in the hippocampal region at 7, 14 and 30 DAI. (C–H) Representative images of the hippocampal regions, showing the morphology and density of Iba1+ cells (green) and GFAP+ cells (red) in not transplanted (C, E, and G) and transplanted (D, F and H) brain hemispheres at 7, 14 and 30 DAI. Abbreviations: cc  =  corpus callosum. Scale bars: 50 µm.

NSCs can act as immunomodulators in pathological, inflammatory brain environments [20], [26], [36]–[38]. We have analyzed both the quantitative reduction and morphological changes in reactive microglial Iba1+ cells and GFAP+ astrocytes, in post-ischemic hippocampal area after IhNSC-Ps transplantation. As shown in Fig. 7A, B, 1 week after transplantation, the inflammatory reaction was significantly reduced in the transplanted hemisphere as compared to the controlateral one (Fig. 7C, D), which displayed an inflammation pattern comparable to that observed earlier on, i.e. at 3DAI (Figure S1D–F). Indeed the fraction of Iba1+ cells in the ipsilateral emisphere amounted to 9.45±3.06% of total cells in the area as compared to 14,71±3.23% in the controlateral one (n = 3). Similarly GFAP+ cells in the ipsilateral side were 8.63±1.07% of the total cells, much less than the 13.13±2.69% detected controlaterally. This difference was even more striking at a later time, i.e. at 14 DAI (Fig. 7E and F), when the inflammatory reaction induced by the lesion is known to reach its peak: the fractions of Iba1+ cells were 10.57±2.64% and 29.36±7.8% and those of GFAP+ cells 6.9±2% and 31.65±3.11% in the transplanted and control hemispheres, respectively (n = 3 each). Moreover, the morphology of both Iba1+ and GFAP+ cells appeared to shift from ameboid and globular, typical of a reactive phenotype, to branching and star-shaped, like in resident cells. At 1 month after lesion inflammation had subsided in both sides and, no significant differences were detectable between the two hemispheres (Fig. 7A and B, G and H).

It is worth noting that immunosuppression by cyclosporine, be it administered transiently or even continuously, did not affect inflammatory response in lesioned animals, as assessed by immunofluorescence using anti-Iba1 antibody (not shown).

## Discussion

### Cell survival and migration

In the adult brain, ischemia and brain trauma increase neurogenesis in the SVZ and migration of newly generated NSC-derived progenitors to the sites of injury [39], [40]; however this self-repair process is limited [7], so that cell therapy through transplantation of exogenous neural cells has been envisioned as a candidate therapeutic approach [13], [41]–[43]. The present study aimed at defining the capacity of IhNSCs for engraftment, migration and differentiation in the adult brain following and ischemic lesion, in view of their perspective use in modeling neural transplantation of human neural cells in ischemia and as a source of cells for cell replacement therapy.

IhNSCs can generate significant percentages of mature neurons and oligodendrocytes *in vitro* [14]. Thus, we investigated their ability to do the same *in vivo*, in the brain of adult rats which suffered transient global ischemia, causing a widely distributed brain injury that primarily affects the neocortical and the hippocampal CA1 layers [22], [23]. As the survival of fetal neural tissue is markedly impaired when grafted within the severely lesioned area in ischemic lesion, but not in the surrounding area [44], the latter was chosen as elective site of injection of the cells. More specifically, IhNSC-Ps were transplanted in the posterior periventricular region, below the neocortical layers and in the hippocampal fissure below the damaged CA1 region. The first evidence to emerge was that the survival rate of the grafts was higher when cells were injected into the posterior periventricular region next to the cc (81.2% of cc transplanted animals, n = 13/16) than in hf (53.3% of hf transplanted animals, n = 8/15). Also, quite remarkably, IhNSC-Ps which were transplanted in the cc showed a preferential tropism for the lesioned cortex, while IhNSC-Ps injected into the hf migrated primarily to colonize the NSC niche in the DG (SGZ). This seems to suggest the existence of differential, locoregional instructive cues in these brain regions, although this phenomenon shall require a specific investigation to be fully unraveled, also considering the proximity of the two injection sites.

We found that, even upon the transient immunosuppression conditions used here, IhNSC-Ps integrated into the cortex, corpus callosum and DG of the hippocampus as early as 14 DAI, also migrating along preferential neurogenic pathways [31], acquiring a typical neuronal morphology. By 30 DAI, IhNSC-Ps injected in the periventricular region were migrating tangentially along the cc, as shown by previous studies [45]. Consistent with this pattern, IhNSCP-derived cells were found in the controlateral hemisphere, diffusely spreading through the white matter, 4 months post implantation. When injected in the proximity of the DG, IhNSC-Ps migrated along the SGZ (30 DAI), possibly attracted by endogenous environmental cues secreted by the activated stem cell niche layer [32]; in accordance with these observations, at the endpoint of the analysis, we detected IhNSCP-derived cells being distributed all along the medial dorsal wall of the SVZ in the 3^rd^–4^th^ ventricle. The evidence above seems to suggest the existence of differential, locoregional instructive cues in the cc and DG, although this phenomenon shall require a full blown study to be fully unraveled.

At least 19% of the grafted human cells survived 1 month after transplantation, a percentage similar to that observed after 4 months. This would suggest a quite stable profile of integration and survival of the transplanted cells over a quite long period. Nonetheless, it is also possible that this apparent stability in the overall number of grafted cells may be the consequence of a dynamic balance between two competing processes, the death of engrafted cells and the birth of new ones through cell proliferation. In fact, it is well known that stroke-associated hypoxia enhances the proliferation of neuronal precursors [40], [46], [47] and, in agreement with this, we documented the sporadic presence of Ki67+ elements among our donor cells.

Only 7 out of 28 of the transplanted control (unlesioned) animals showed appreciable cell engraftment and survival with respect to 37 out of 61 in lesioned animals. Furthermore, in control animals, engrafted cells were mainly localized next to the injection site (not shown). These results are in good agreement with previous studies, showing that both IhNSC-Ps survival and migration are enhanced by the presence of a brain lesion as compared to the healthy CNS tissue [29]. Finally, the transplanted IhNSC-Ps were not tumorigenic, in accordance with our earlier findings in SCID mice [14]. Our findings are also consistent with studies carried out with primary cultures of human progenitors [40], [48], [49] and provide the initial evidence that neural progenitors that are continuously produced by a renewable source of hNSCs can undergo targeted migration to different areas in the adult brain affected by a global ischemic lesion.

### IhNSC-Ps proliferation and differentiation

Others and us have previously shown that NSCs undergo prevalent glia differentiation after transplantation in neurodegenerative disease animal models such as metachromatic leukodystrophy (MLD) [20], focal demyelination [24] and multiple sclerosis (MS) [38]. In order to enhance the neuronogenic potential of IhNSCs, in this study we transplanted neural progenitors derived from IhNSCs (IhNSC-Ps) which were pre-committed to differentiation in vitro and cultured at 5% oxygen, a condition approximating the physiological range of oxygen in the SVZ and DG [50]. As early as 1 month from transplantation into the brain of ischemic adult rats, clusters of HuN+ cells expressed markers of early neuronal progenitors (Dcx+ and NCAM+), and we also identified HuN+/β-Tub+ and HuN+/MAP2+ neurons with ramificated morphology and typically star-shaped HuN+/GFAP+ astrocytes. The expression of these markers was maintained at 4 months from transplantation when IhNSC-Ps appeared widely distributed in the corpus callosum and cortex, where we could detect HuN+ cells bearing GABAergic or Glutamatergic phenotypes, consistent with their physiological prevalence in these brain areas and with the pattern of *in vitro* differentiated hNSCs and IhNSCs [14], [34]. To note, the synthesis of GABA by newborn neurons and active cortical neurogenesis by resident progenitors of layer 1 have been recently shown to be a fundamental requisite to restore neuronal function after stroke [33], [51]. Intriguingly, we also found sporadic MAP2+/humanKi67+ cells at 3 months post ischemia, which completely disappear at 4 months, indicating that at least a fraction of IhNSC-Ps undergo transient short–term proliferation, also favoured by the local specific environmental cues [33]. Most importantly, our findings show the expression of both GABA and Glutamate neurotransmitters by a renewable source of human cells transplanted in a lesioned adult rodent brain.

### IhNSC-Ps are not immunogenic under transient immunosuppression treatment

A wide array of studies have shown that NSCs are not susceptible to immunological rejection [15]–[18] even when transplanted in animal models like EAE, characterized by a constitutively activated immunological response [25], [26]. Notably, others and us have documented the ability of NSCs to somewhat modulate or even dampen immunological response upon transplantation [26], [36], [38], [43]. This phenomenon may, in fact, participate in the low immunogenic response that these cells seem to elicit in the CNS. Notwithstanding, it is also true that some level of immune surveillance is maintained in the adult brain upon NSCs engraftment, which explains the widespread need to use immune suppression [18] in experimental and clinical intracerebral transplantation [15], [17].

The succesful use of transient immunosuppression described here, supports the twofold notion of limiting toxicity in an experimental model plagued by high animal mortality and of preventing the bias introduced by the known neuroprotective effects of cyclosporine following hypoxia-ischemia [52], and proposes a suitable milder approach to immunosuppression for the prospective use hNSCs for clinical purposes. That the discontinuous treatment with cyclosporine does not affect integration of transplanted cells in most of the brain regions, which to all effects emerge as immunoprivileged when considering hNSCs, is in good accordance with most recent findings [18]. It should also be noted that the rate of survival of our transplanted IhNSC-Ps appeared more prominent than that observed in xenografts of embryonic human neural precursor cells [53], transplants of fetal tissue into patients with Parkinson's Disease [15] or of adult human NPCs in ischemic rats [17].

### Generation of mature neurons and reduction of the inflammation by IhNSC-Ps in ischemia

Our ultrastructural analysis determined the full maturation of IhNSCs progeny by detecting the presence of newly established synaptic junctions between rat axonal terminals and IhNSC-Ps progeny dendritic spines in the CA1 layer, 4 months after transplantation. This is consistent with previous observations, showing the ability of IhNSCs to generate post-synaptic structures and to fire spontaneous action potentials in culture [14] and is further supported by the detection of GFP labeled axons enveloped by a multilayered myelin structure. Given the prolonged timing required by human neural progenitors to mature in vivo, analysis at further time points could provide additional details on the functional integration of transplanted cells in the damaged neuronal circuitry. Unfortunately, the age and size of adult rats, combined with the dysplastic condition of the ischemic brain tissue allowed neither electrophysiological studies, nor an ultrastructural investigation beyond the 4 month end/point.

Besides neurodegeneration *per se*, one of the hallmarks characterizing most neurodegenerative disorders like stroke, AD, PD, ALS, MLD [37], is the development of an inflammatory environment, which can contribute to tissue damage. Recent studies have shown that NSCs may also exert their therapeutic potential through an immunomodulatory action [26], [36], [38]. Our study reports that transplantation of IhNSCs can effectively decrease reactive astrogliosis and dumpen microglial activation in the injuried areas. This effect occurred exclusively in the transplanted regions and was most prominent at 15 days from transplantation, when the inflammatory reaction appeared to reach its nadir. There was an obvious effect on the state of activation of microglia, whose cells shifted from the activated, macrophagic-amoeboid phenotype to the resting, stellate one, with a concomitant shift of astrocytes from fibrotic and globular to star-shaped and long-branching in the transplanted areas.

### Conclusion

Transient global ischemia is a commonly accepted model of vascular dementia, since it resembles the pathological features of Alzheimer's Disease. In this view, the findings presented in this manuscript lend to the idea of using IhNSCs as a suitable tool to model transplantation of hNSCs in pre-clinical settings. This is particularly relevant in view of the fact that the first phase I clinical trial exploiting cell therapy has been authorized and is currently underway. The trial uses non-immortalized neural cells similar to those described here, which may thus be considered for a prospective use in clinical settings. This is particularly true, considering the suitable migration and differentiation pattern of our IhNSCs in the ischemic brain, their negligible rejection, their ability to establish synaptic interaction with host cells and their capacity to generate appropriate neurotransmitter phenotypes in ischemia target areas, such as the hippocampus and cortex. The ability of transplanted IhNSC-Ps to dampen reactive astrogliosis and microglia activation provide an extra positive element when considering hNSCs for therapeutic purposes in neurodegenerative disorders.

## Supporting Information

Figure S1 Analysis of the lesioned brain at 3DAI. (A–C) Hematoxylin-eosin showing the pyknotic nuclei present in the lesioned (A, arrows) respect to the control cortex (B), and in the lesioned CA1 layer (C). (D–E) Microglial (Iba1+, D) and astroglial (GFAP+, E) reaction in the hippocampal region of lesioned animals at 3DAI. (F) Quantification of pyknotic nuclei in the CA1 layer and of Iba1+ and GFAP+ cells in the hippocampal region. Scale bar: A and B: 50 µm, C: 5 µm, D and E: 75 µm.
(5.38 MB TIF)

Figure S2 *In vitro* differentiation of IhNSC. (A–E) IhNSC-P used for transplantation contained early neuronal progenitors (Dcx+, A and NCAM+, B), neurons (β-Tub+, C and MAP2+, D), astrocytes (GFAP+, C), oligodendrocytes (GalC+, E) and a percentage of residual proliferating cells (Ki67+, A, B and D). (F) Quantification of the neural cell lineages in IhNSC-P. Scale bars: A–E: 10 µm.
(5.00 MB TIF)

Figure S3 Experimental design. (A) Schematic representation showing the experimental plan with transplanted animals undergoing transient or constitutive immunosuppression. Healthy not transplanted animals (n = 4) have been excluded. (B) Table showing the numerosity of the transplanted animal groups. Abbreviations: cc: corpus callosum, hf: hippocampal fissure, AP: anteroposterior, L: lateral, DV: dorsoventral.
(9.30 MB TIF)
