# Supplementary figures and images for: Long-Term Survival of Human Neural Stem Cells in the Ischemic Rat Brain upon Transient Immunosuppression
Source: PLoS One. 2010 Nov 19;5(11):e14035. doi: 10.1371/journal.pone.0014035 (PMC2988794; doi:10.1371/journal.pone.0014035)

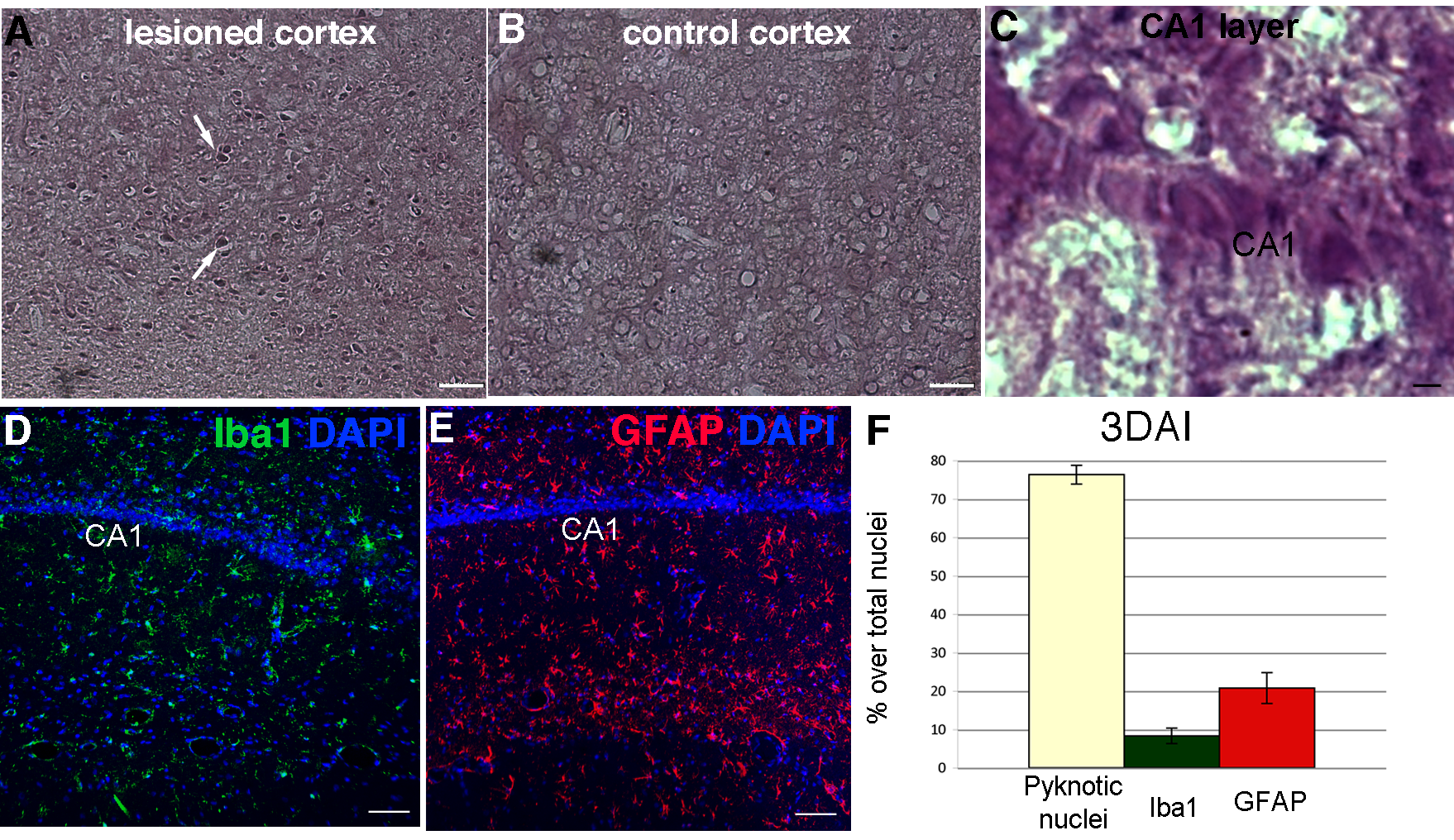

Supplement: Figure S1 — Analysis of the lesioned brain at 3DAI. (A–C) Hematoxylin-eosin showing the pyknotic nuclei present in the lesioned (A, arrows) respect to the control cortex (B), and in the lesioned CA1 layer (C). (D–E) Microglial (Iba1+, D) and astroglial (GFAP+, E) reaction in the hippocampal region of lesioned animals at 3DAI. (F) Quantification of pyknotic nuclei in the CA1 layer and of Iba1+ and GFAP+ cells in the hippocampal region. Scale bar: A and B: 50 µm, C: 5 µm, D and E: 75 µm. (5.38 MB TIF) [file pone.0014035.s001.tif]

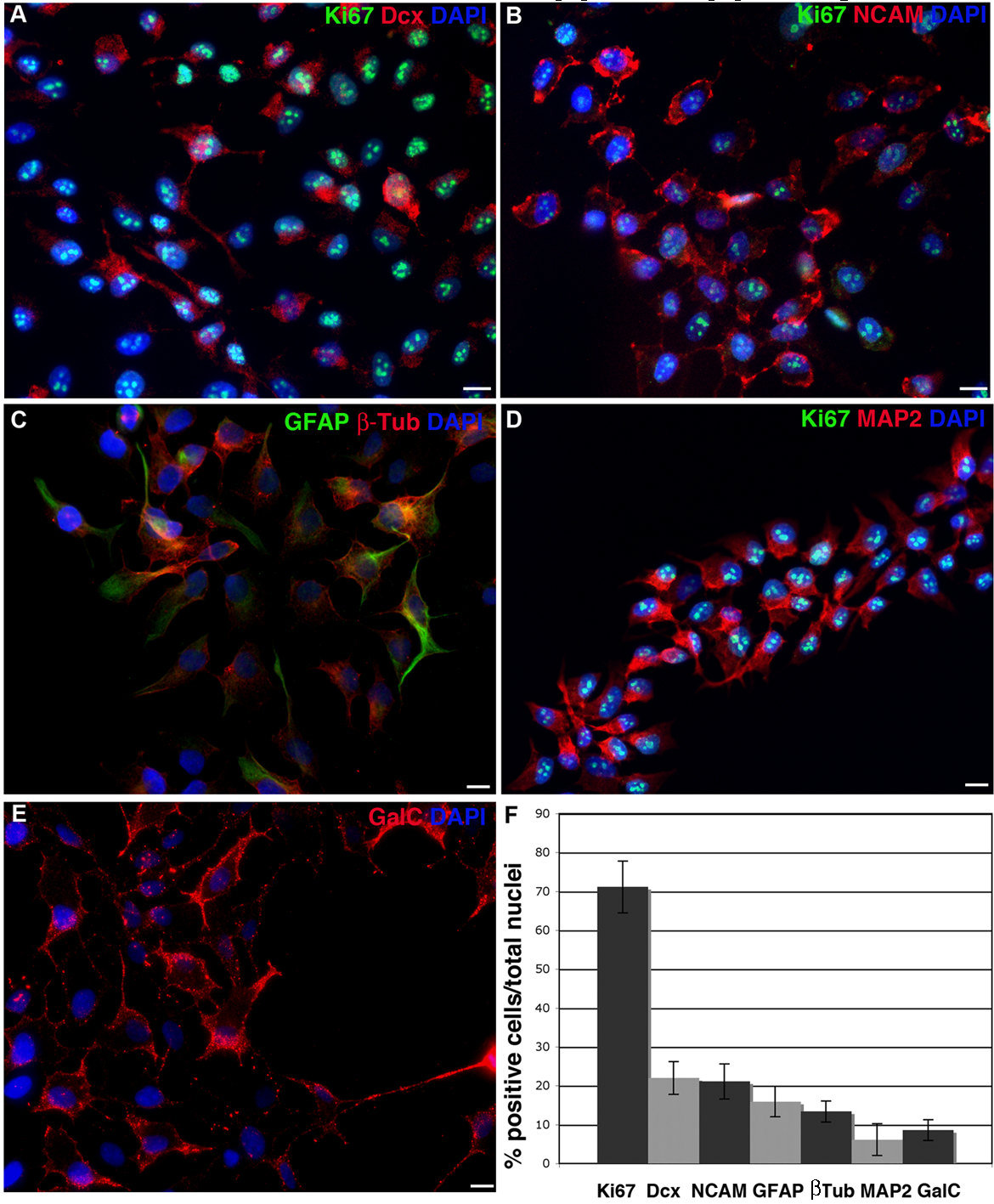

Supplement: Figure S2 — In vitro differentiation of IhNSC. (A–E) IhNSC-P used for transplantation contained early neuronal progenitors (Dcx+, A and NCAM+, B), neurons (β-Tub+, C and MAP2+, D), astrocytes (GFAP+, C), oligodendrocytes (GalC+, E) and a percentage of residual proliferating cells (Ki67+, A, B and D). (F) Quantification of the neural cell lineages in IhNSC-P. Scale bars: A–E: 10 µm. (5.00 MB TIF) [file pone.0014035.s002.tif]

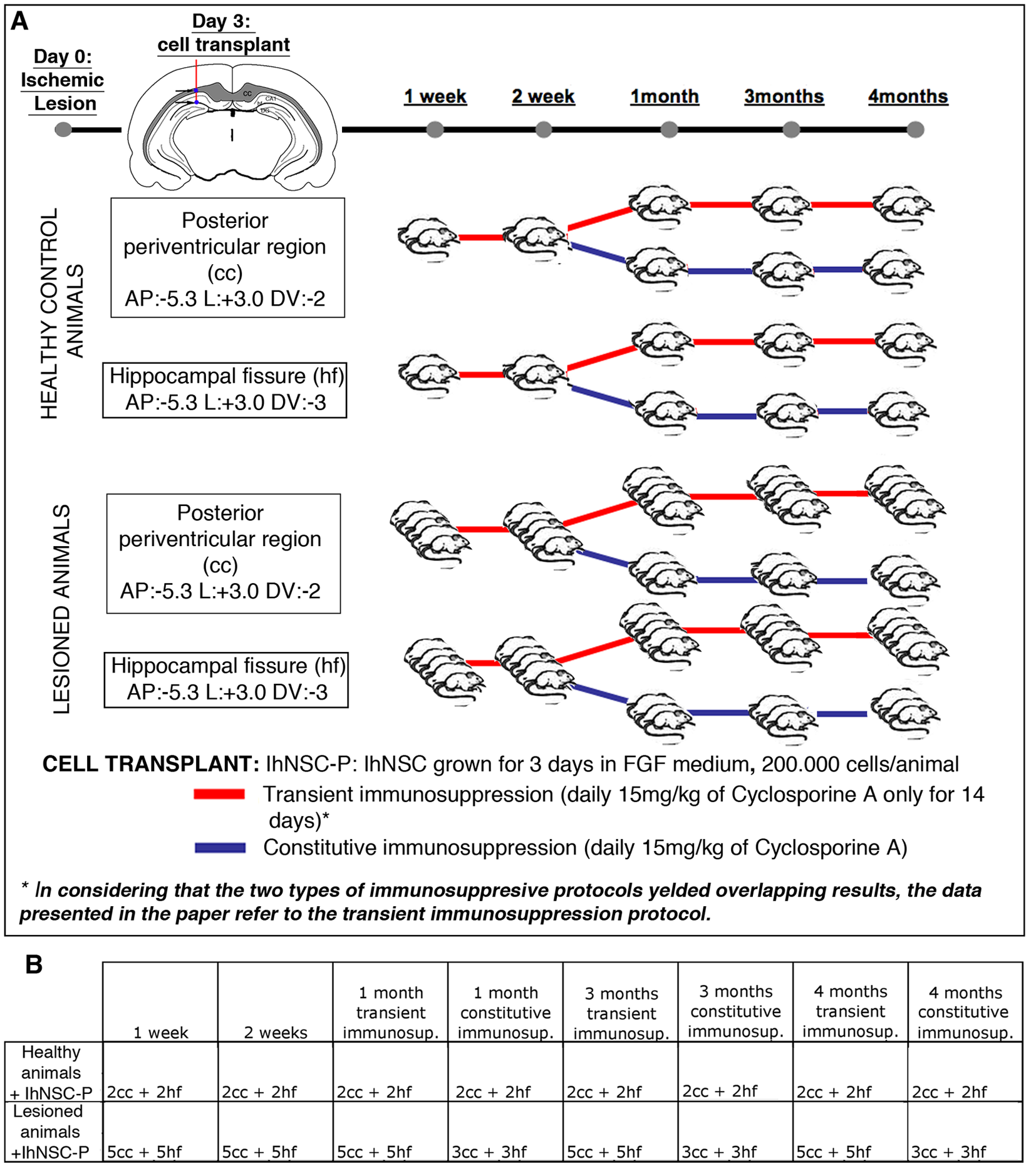

Supplement: Figure S3 — Experimental design. (A) Schematic representation showing the experimental plan with transplanted animals undergoing transient or constitutive immunosuppression. Healthy not transplanted animals (n = 4) have been excluded. (B) Table showing the numerosity of the transplanted animal groups. Abbreviations: cc: corpus callosum, hf: hippocampal fissure, AP: anteroposterior, L: lateral, DV: dorsoventral. (9.30 MB TIF) [file pone.0014035.s003.tif]
